# Evaluation of Pafolacianine (Cytalux^®^) for Fluorescence-Guided Surgery in Head and Neck Squamous Cell Carcinoma: A Negative Study with Important Clinical Implications

**DOI:** 10.1007/s11307-025-02068-3

**Published:** 2025-12-11

**Authors:** Lucas Mani, Syeda Maria Ahmad Zaidi, Estelle Martin, Carleigh Rose Burns, Abdullah Bin Naveed, Ashtyn McAdoo, Hidenori Tanaka, Eben Rosenthal, Marisa Hom

**Affiliations:** Department of Otolaryngology-Head and Neck Surgery, Vanderbilt University Medical Center, Nashville, TN 37232 USA

**Keywords:** Pafolacianine, Cytalux, Fluorescence-guided surgery, Head and neck squamous cell carcinoma, Folate receptor, Negative study, Precision medicine

## Abstract

**Background:**

Pafolacianine (Cytalux^®^) represents the first FDA-approved tumor-specific fluorescence imaging agent, demonstrating efficacy in ovarian cancer through folate receptor-α (FR-α) targeting. Given the need for improved intraoperative margin assessment in head and neck squamous cell carcinoma (HNSCC), where positive surgical margins occur in 10–30% of cases, we investigated the potential utility of pafolacianine for fluorescence-guided surgery in HNSCC models.

**Objective:**

To evaluate the feasibility of visualizing HNSCC using pafolacianine *in vitro*, *in vivo*, and clinical tissue analysis, with comparison to fluorescence-guided surgery agents that have been successful in patients.

**Methods:**

HNSCC cell lines (FaDu, UMSCC47) were treated with escalating concentrations of pafolacianine (0–500 nM) and assessed for binding at 1 and 24 h. Nude mice bearing HNSCC xenografts (FaDu, UMSCC47) received intraperitoneal injection of pafolacianine (10 nmol) with fluorescence imaging at multiple timepoints. Immunohistochemistry analysis of patient samples (*n* = 8 tumor, *n* = 8 normal) evaluated FR-α and FR-β expression. Panitumumab-IRDye800CW served as a positive control for comparison.

**Results:**

*In vitro* analysis demonstrated minimal pafolacianine binding across all HNSCC cell lines, with fluorescence intensities similar to or lower than the FR-α-negative A549 control cell line. *In vivo* imaging revealed poor tumor localization with mean fluorescence intensity (MFI) of 7.39 (FaDu) and 6.97 (UMSCC47), substantially lower than non-target tissues including skin. Immunohistochemistry analysis showed no statistically significant difference in FR-α expression between tumor and normal tissue (p > 0.05). For comparison, panitumumab-IRDye800CW demonstrated robust tumor targeting with MFI of 32.14 (FaDu) and 14.98 (UMSCC47).

**Conclusions:**

This study demonstrates that pafolacianine exhibits limited utility for fluorescence-guided surgery in HNSCC due to insufficient FR-α expression and poor tumor-to-background contrast. These negative findings provide crucial evidence against the clinical translation of pafolacianine for HNSCC applications and highlight the importance of target expression validation in precision medicine approaches.

**Clinical Relevance:**

Negative studies such as this are essential for evidence-based clinical decision-making, preventing unnecessary resource allocation and potential patient exposure to ineffective interventions. These findings inform the broader fluorescence-guided surgery field and support continued investigation of alternative targeting strategies for HNSCC.

**Supplementary Information:**

The online version contains supplementary material available at 10.1007/s11307-025-02068-3.

## Introduction

Fluorescence-guided surgery (FGS) has emerged as a transformative approach in oncological surgery, offering real-time visualization of tumor tissue to improve surgical precision and patient outcomes [1]. The recent FDA approval of pafolacianine (Cytalux^®^), the first tumor-specific fluorescence imaging agent, represents a significant milestone in the field of precision surgery [2]. Initially approved for ovarian cancer imaging based on folate receptor-α (FR-α) overexpression [3], pafolacianine has demonstrated clinical utility in improving surgical outcomes, with 26% of lung cancer patients showing improved surgical decision-making following its implementation [4]. The opportunity to repurpose this agent for other cancer types represents a huge opportunity to improve patient care with limited regulatory burden.

The clinical need for improved intraoperative guidance is particularly acute in head and neck squamous cell carcinoma (HNSCC), where achieving negative surgical margins remains one of the most critical determinants of patient prognosis [5]. The anatomical complexity of the head and neck region, combined with the proximity of vital structures, contributes to positive margin rates of 10–30% in HNSCC resections—among the highest across all cancer types [6]. Positive surgical margins necessitate adjuvant chemoradiation therapy, significantly increasing healthcare costs and patient morbidity while compromising long-term survival outcomes [7]. This clinical challenge underscores the urgent need for more accurate methods of intraoperative tumor visualization in HNSCC. Since the clinical development of new imaging agents is expensive without the same fiscal return as therapeutic agents, the repurposing of these agents is an excellent opportunity to improve care across tumor types.

The biological rationale for investigating pafolacianine in HNSCC stems from the established role of folate receptors in cancer biology. FR-α is a glycosylphosphatidylinositol-anchored membrane protein that facilitates cellular folate uptake and has been identified as overexpressed in various solid tumors [8]. The success of pafolacianine in ovarian cancer, where FR-α overexpression is well-documented, suggested potential applicability to other cancer types with similar receptor expression patterns [3]. Pafolacianine functions through a folate analogue conjugated to an indocyanine green-like near-infrared fluorophore via a vinyl ether bridge, enabling specific targeting of FR-α-expressing cells with peak fluorescent emission at 796 nm [9].

Our hypothesis was that pafolacianine would demonstrate sufficient FR-α-mediated binding and tumor localization in HNSCC models to warrant clinical investigation. However, as detailed in the following sections, our evaluation revealed negative results across all experimental approaches which we consider will provide value as a caution against additional testing in this cancer type [10].

## Materials and Methods

### Study Design and Ethical Considerations

This preclinical study was designed as a comprehensive evaluation of pafolacianine efficacy in HNSCC models through multiple complementary experimental approaches. The study protocol incorporated *in vitro* binding assays, *in vivo* xenograft imaging, and immunohistochemical analysis of clinical specimens to provide definitive evidence regarding the potential utility of pafolacianine for HNSCC fluorescence-guided surgery. All animal experiments were conducted under protocols approved by the Vanderbilt University Medical Center Institutional Animal Care and Use Committee, ensuring adherence to the highest standards of animal welfare and scientific rigor.

### Clinical Specimen Collection and Processing

Human tissue specimens were obtained through the Head and Neck Cancer Tissue Repository and Clinical Database (NCT00898638) under Institutional Review Board-approved protocols at Vanderbilt University Medical Center. The study cohort comprised 16 total specimens: 8 tumor samples and 8 matched normal tissue controls from patients with HNSCC affecting various anatomical subsites. The tumor distribution included laryngeal carcinomas (*n* = 2), oropharyngeal carcinoma (*n* = 1), parapharyngeal wall carcinoma (*n* = 1), and oral cavity carcinomas (*n* = 4), providing representation across the major HNSCC anatomical subsites.

All patients provided written informed consent prior to tissue collection, and specimens were de-identified in accordance with Health Insurance Portability and Accountability Act (HIPAA) guidelines. The study was conducted in full compliance with the Declaration of Helsinki principles for human research. Tissue specimens were processed using standard formalin-fixed, paraffin-embedded (FFPE) protocols to ensure optimal preservation of antigenic epitopes for subsequent immunohistochemical analysis.

### Immunohistochemical Analysis

Immunohistochemical staining was performed at the Vanderbilt Translational Pathology Shared Resource core laboratory using standardized protocols optimized for FFPE tissue sections. Primary antibodies targeting FR-α (ThermoFisher Scientific, catalog #PA5-24,186) and FR-β (ThermoFisher Scientific, catalog #MA5-26,933) were applied using validated dilutions and incubation conditions. Staining intensity and distribution were evaluated by a board-certified pathologist blinded to clinical outcomes using a standardized scoring system.

The immunohistochemical scoring methodology incorporated both staining intensity (0–3 scale) and percentage of positive cells (0–100%) to generate composite scores ranging from 0–9. Scores were categorized as low (0–2), intermediate (3–5), or high (6–9) expression levels. Both epithelial and stromal compartments were evaluated separately to provide comprehensive assessment of receptor expression patterns within the tumor microenvironment.

### Cell Culture and Characterization

A comprehensive panel of HNSCC cell lines was selected to represent the biological diversity of head and neck cancers. The cell lines included FaDu (hypopharyngeal origin, ATCC #HTB-43), UMSCC1 (floor of mouth origin, Millipore Sigma #SCC070), UMSCC17B (metastatic neck node origin, Millipore Sigma #SCC075), UMSCC47 (lateral tongue origin, Millipore Sigma #SCC071), SCC25 (tongue origin, ATCC #CRL-1628), and SCC9 (tongue origin, ATCC #CRL-1629). Control cell lines included A549 (lung adenocarcinoma, ATCC #CCL-185) as an FR-α-negative control and KB cells (oral carcinoma, ATCC #CRL-3596) as a positive control.

Cell culture conditions were optimized for each cell line based on ATCC recommendations. UMSCC cell lines were maintained in Dulbecco's Modified Eagle Medium (DMEM, Gibco #11,965,092) supplemented with 10% fetal bovine serum (FBS, Biotechne #S11150-NOV), 1% penicillin–streptomycin (Gibco #15–070-063), and 1% non-essential amino acids (Gibco #11,140,050). FaDu, SCC25, and SCC9 cells were cultured in DMEM/F-12 medium (Gibco #11,320,033) with 10% FBS, 1% penicillin–streptomycin, and 400 ng/mL hydrocortisone (Stemcell Technologies #NC1062339). A549 cells were maintained in F-12 K medium (Gibco #21,127,022) with 10% FBS, while KB cells were cultured in Eagle's Minimum Essential Medium (Gibco #11,095,080) with 10% FBS.

All cell cultures were maintained at 37 °C in a humidified atmosphere containing 5% CO₂. Cell lines underwent routine authentication using short tandem repeat (STR) analysis and were regularly screened for mycoplasma contamination to ensure experimental validity. Cell passage numbers were carefully tracked, and experiments were conducted using cells within validated passage ranges to maintain phenotypic stability.

### Fluorescent Agent Preparation and Characterization

**Fig. 1:**
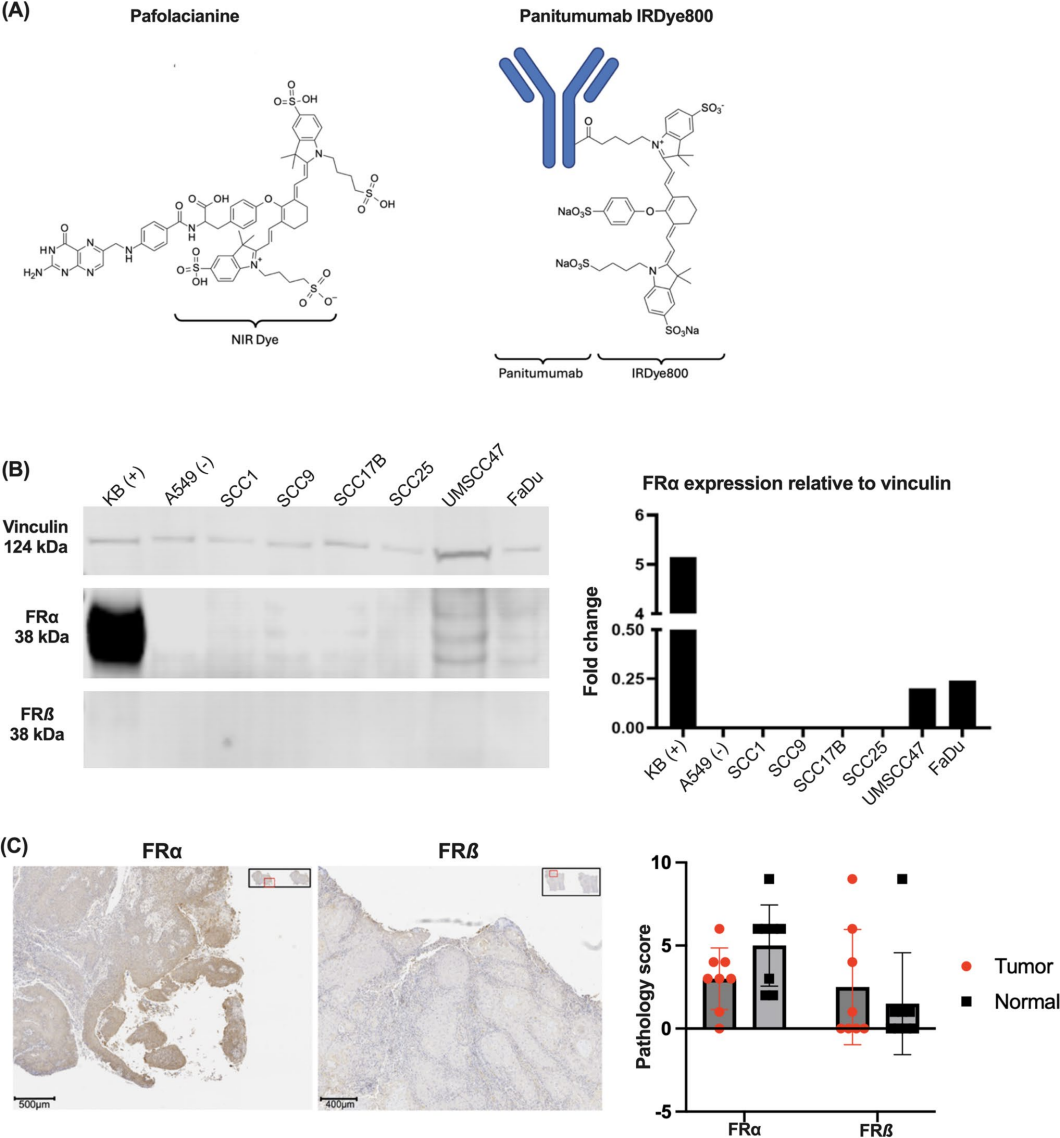
Initial analysis of pafolacianine feasibility in HNSCCs. **(A)** Structure of pafolacianine (left, peak fluorescent emission at 796 nm) compared to Panitumumab-IRDye800 (right, peak fluorescent emission at 792 nm). **(B)** Western blot analysis of FRα (38 kDa) and FRβ (30 kDa) relative abundances in control and HNC cell lines Vinculin (124 kDa) is included as a loading control. **(C)** Pathological analysis of FRα/β in human HNSCC tumor samples, with graphical comparison of FRα and FRβ in tumor tissue versus normal tissue in human patients

Pafolacianine (Cytalux^®^) was provided by On Target Laboratories (West Lafayette, IN, USA) as a sterile solution (3.2 mg/1.6 mL) for investigational use. The agent consists of a folate analogue conjugated to an indocyanine green-like near-infrared fluorophore via a vinyl ether bridge (Fig. 1A), with peak fluorescent emission at 796 nm. Stock solutions were prepared according to manufacturer specifications and stored at 4 °C protected from light to maintain fluorescent stability.

Panitumumab-IRDye800CW was synthesized in-house using established protocols. Briefly, clinical-grade panitumumab (Vectibix^®^, Amgen) was conjugated to IRDye800CW using a Li-Cor Protein Labeling Kit (Li-Cor #929–70,020) following manufacturer instructions. The conjugation reaction was performed at room temperature for 4 h with 3 molar equivalents of fluorophore, protected from light throughout the process. Excess fluorophore was removed using Zeba™ Spin Desalting Columns (ThermoFisher #89,891), and the final product was characterized for concentration (1.0 mg/mL) and dye-to-protein ratio (0.89) using spectrophotometric analysis.

### *In Vitro* Binding Assays

Quantitative assessment of pafolacianine binding was performed using standardized fluorescence-based assays optimized for high-throughput analysis. Cells were seeded in 96-well black-walled, clear-bottom plates (ThermoFisher #164,588) at a density of 2 × 10^3^ cells per well and cultured overnight to achieve 70–80% confluence. This seeding density was selected to ensure adequate cell numbers for fluorescence detection while avoiding confluence-related artifacts.

Following overnight incubation, culture medium was replaced with fresh medium containing serial dilutions of pafolacianine (0–500 nM) or panitumumab-IRDye800CW (0–10,000 nM). The concentration ranges were selected based on previous literature and preliminary optimization studies to encompass both sub-therapeutic and saturating concentrations. Cells were incubated with fluorescent agents for defined time periods (1, 8, 24, and 72 h) at 37 °C in a humidified 5% CO₂ atmosphere.

At designated timepoints, cells were washed twice with phosphate-buffered saline (PBS) to remove unbound fluorescent agent, and fluorescence intensity was measured using an Odyssey CLx imaging system (Li-COR Biosciences). Fluorescence measurements were performed at 800 nm wavelength with standardized acquisition parameters across all samples. Mean fluorescence intensity (MFI) values were calculated for each well and normalized to cell number to account for potential variations in cell density.

### *In Vivo* Xenograft Studies

Animal studies were conducted using female nude mice (Charles River, #24,102,241) housed under specific pathogen-free conditions in accordance with Institutional Animal Care and Use Committee guidelines. Mice were quarantined for one week following arrival to ensure health status and minimize stress-related variables. All procedures were performed under isoflurane anesthesia to ensure animal welfare and experimental consistency.

Xenograft tumors were established through subcutaneous injection of 1 × 10⁷ cells in 100 μL of a 1:1 mixture of sterile Dulbecco's PBS (ThermoFisher #14–080-055) and Matrigel (ThermoFisher #CB-40234A) into the left flank. Two HNSCC cell lines were selected for *in vivo* studies: FaDu and UMSCC47, representing different anatomical origins and biological characteristics. Tumor growth was monitored using caliper measurements, and experiments were initiated when tumor volumes reached approximately 150 mm^3^ to ensure adequate tumor size for imaging while minimizing animal discomfort.

Fluorescent agent administration was performed via intraperitoneal injection to ensure systemic distribution and minimize injection site artifacts. Experimental mice received a single dose of 10 nmol pafolacianine, while control animals received 75 μmol panitumumab-IRDye800CW based on established protocols from previous studies. The timing of imaging was optimized based on pharmacokinetic considerations, with pafolacianine imaging performed at 2, 24, 48, and 72 h post-injection, and 24 h was determined to be the most optimal time for intra-tumoral drug visualization. Panitumumab-IRDye800CW imaging was conducted at 72 h post-injection based on established optimal timing.

Fluorescence imaging was performed using a PEARL Trilogy small-animal imaging system (Li-COR Biosciences) with standardized acquisition parameters. Animals were anesthetized with isoflurane and positioned consistently for imaging to ensure reproducible measurements. Both dorsal and ventral imaging positions were acquired to provide comprehensive assessment of fluorescent agent distribution.

Following *in vivo* imaging, animals were euthanized at predetermined timepoints, and comprehensive tissue harvesting was performed. Collected tissues included tumor, skin, muscle, liver, kidney, spleen, colon, heart, and lung samples to assess biodistribution patterns and calculate tissue-specific mean fluorescence intensity (MFI). Ex vivo fluorescence quantification was performed immediately following tissue harvest to minimize fluorescence decay artifacts. MFI was computed by drawing regions of interest (ROI) around the resected specimens and normalized to the fluorescence of the respective plate, which was taken as background signal.

### Statistical Analysis and Data Management

All statistical analyses were performed using GraphPad Prism software version 9.0 with predetermined significance thresholds and appropriate statistical tests for each data type. Sample size calculations were performed a priori based on effect sizes observed in preliminary studies and published literature to ensure adequate statistical power for detecting meaningful differences.

For *in vitro* binding studies, concentration–response curves were analyzed using nonlinear regression analysis to determine IC₅₀ values and binding parameters. Time-course data were analyzed using two-way analysis of variance (ANOVA) with Bonferroni post-hoc testing for multiple comparisons. *In vivo* imaging data were analyzed using unpaired Student's t-tests for comparisons between treatment groups, with Welch's correction applied when variances were unequal.

Immunohistochemical scoring data were analyzed using Mann–Whitney U tests for non-parametric comparisons between tumor and normal tissue groups. Correlation analyses were performed using Spearman's rank correlation coefficient to assess relationships between different receptor expression patterns. All statistical tests were two-tailed with significance defined as *p* < 0.05. Effect sizes were calculated and reported alongside p-values to provide comprehensive assessment of biological significance.

Data management followed Good Laboratory Practice principles with comprehensive documentation of all experimental procedures, raw data files, and analysis protocols. All primary data were stored in secure, backed-up databases with appropriate access controls to ensure data integrity and reproducibility. Quality control measures included regular calibration of imaging equipment, validation of reagent lots, and inclusion of appropriate positive and negative controls in all experiments.

## Results

### Folate Receptor Expression Analysis in Clinical HNSCC Specimens

Immunohistochemical (IHC) analysis of clinical HNSCC specimens revealed limited and heterogeneous expression of folate receptors across the study cohort. Quantitative assessment of FR-α expression demonstrated no statistically significant difference between tumor tissue (median score: 2.5, interquartile range: 1.0–4.0) and normal adjacent tissue (median score: 2.0, interquartile range: 1.0–3.5; *p* = 0.42, Mann–Whitney U test). Similarly, FR-β expression showed no significant difference between tumor (median score: 1.5, interquartile range: 0.5–3.0) and normal tissue (median score: 1.0, interquartile range: 0.5–2.5; *p* = 0.38) (Fig. 1C). The distribution of expression scores revealed considerable heterogeneity within the tumor cohort, with 62.5% of tumor samples (5/8) demonstrating low expression (scores 0–2), 25% showing intermediate expression (scores 3–5), and only 12.5% exhibiting high expression (scores 6–9) for FR-α. This heterogeneous expression pattern contrasts markedly with the uniform high expression typically observed in ovarian cancer specimens, where pafolacianine has demonstrated clinical efficacy.

Correlation analysis between FR-α and FR-β expression revealed a weak positive correlation (Spearman's ρ = 0.31, *p* = 0.23), suggesting independent regulation of these receptor subtypes in HNSCC. Notably, even specimens with the highest FR-α expression scores (epithelial score = 6, stromal score = 0) represented isolated cases within the broader cohort, indicating that high FR-α expression is not a consistent feature of HNSCC biology.

The anatomical distribution of FR expression showed no clear pattern across different HNSCC subsites, with laryngeal, oropharyngeal, and oral cavity tumors demonstrating similarly low and variable expression levels (Table 1). This finding suggests that the limited FR-α expression observed is not specific to particular anatomical locations but rather represents a general characteristic of HNSCC biology.

**Table 1:** Oncologic features and respective FR- α and FR- β expression in head and neck patient samples

Age (years)/Sex	TNM	Primary tumor size (cm)	Positive lymph nodes	ID*	Primary site	Subsite	FR- α		FR- β	
							Epithelial	Stromal	Epithelial	Stromal
51, Female	T2N0Mx	2.2	0	1N	Oropharynx	Uvula	9	4	0	0
				1T	Oral cavity	Alveolar ridge	6	0	0	0
74, Male	T4aN0Mx	8.4	0	2N	Oropharynx	Uvula	6	0	0	0
				2T	Oral cavity	Floor of mouth	3	0	0	0
49, Male	T4aN2bMx	5.2	4	3N	Oral cavity	Tongue	6	0	0	0
				3T	Oral cavity	Tongue	3	0	0	0
76, Male	T4aN3bMx	6.8	10	4N	Para-pharyngeal	Para-pharyngeal wall	6	6	1	6
				4T	Larynx	Larynx	4	6	0	6
66, Male	T4aN2cMx	6.9	3	5N	Larynx	Larynx	3	1	0	1
				5T	Larynx	Larynx	1	0	0	0
69, Male	T2N1Mx	2.3	1	6N	Oral cavity	Tongue	6	0	1	1
				6T	Oral cavity	Tongue	4	0	1	0
65, Female	T3N3bMx	3.6	3	7N	Oral cavity	Tongue	2	0	1	0
				7T	Oral cavity	Tongue	0	0	4	0
51, Male	T4aN3bMx	3.4	5	8N	Oral cavity	Floor of mouth	3	0	9	6
				8T	Oral cavity	Buccal	2	2	9	6

*N normal sample, *T* tumor sample

### *In Vitro* Pafolacianine Binding Studies

**Fig. 2:**
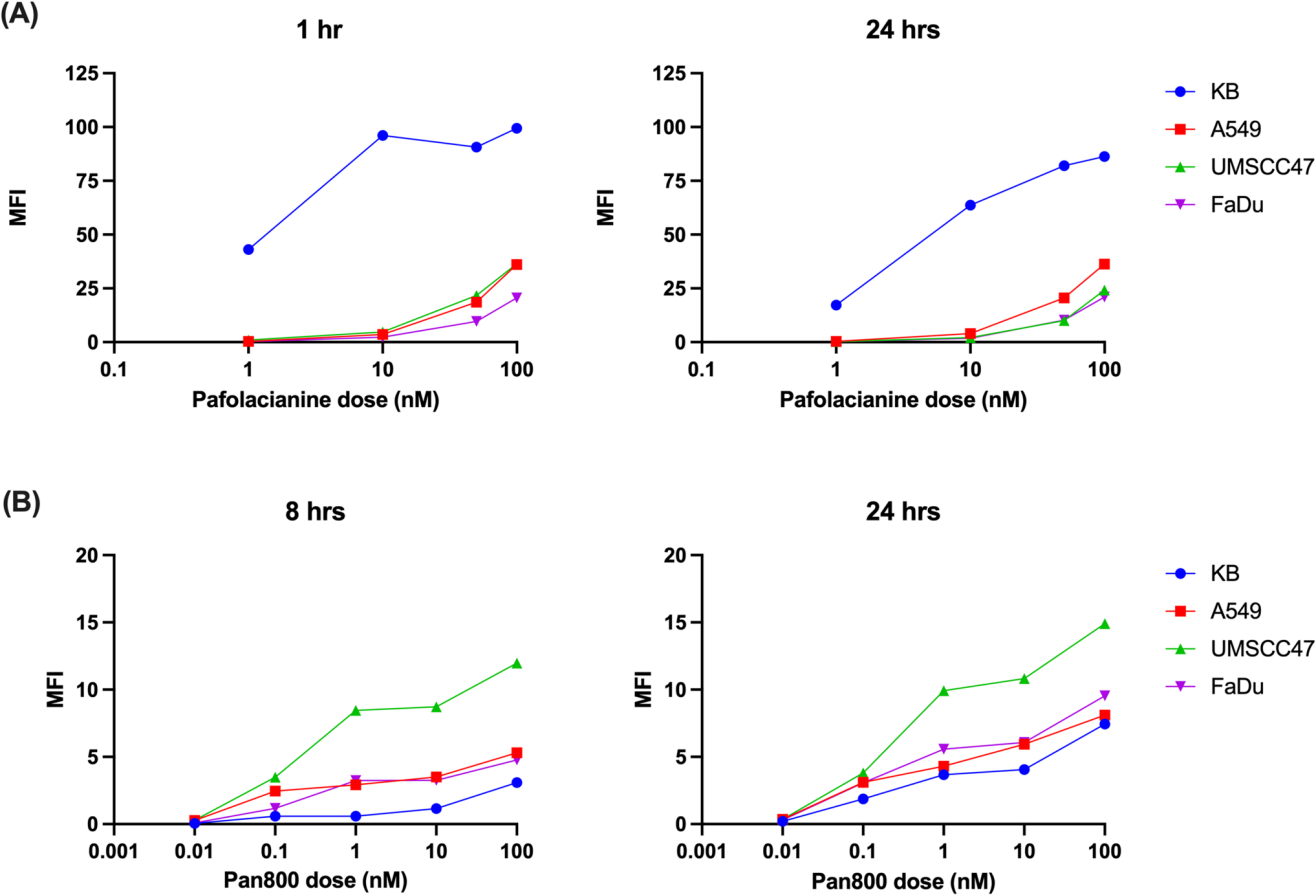
*In vitro* binding of pafolacianine and Pan800 in KB (blue), A549 (red), UMSCC47 (green) and FaDu (pink). **(A)** Average (*n* = 2) MFI of pafolacianine after 1 and 24 h of incubation. **(B)** Average (*n* = 2) MFI of Pan800 after 8 and 24 h of incubation. MFI was calculated by measuring the mean fluorescence of each well and dividing by the area of the well

Comprehensive *in vitro* binding studies across multiple HNSCC cell lines demonstrated consistently poor pafolacianine uptake compared to established positive controls. Dose–response analysis revealed minimal fluorescence accumulation across all tested HNSCC cell lines at both 1-h and 24-h timepoints. Mean fluorescence intensity values for pafolacianine binding are shown in Fig. 2A. These values were comparable to or lower than the FR-α-negative A549 control cell line indicating that the observed fluorescence likely represents non-specific binding rather than FR-α-mediated uptake. In contrast, the KB positive control cell line demonstrated significantly higher pafolacianine binding.

Time-course analysis revealed no significant increase in pafolacianine binding over extended incubation periods, with 24-h values remaining similar to 1-h measurements across all HNSCC cell lines. This finding contrasts with the expected pattern of receptor-mediated endocytosis, where specific binding increases over time. Comparative analysis with panitumumab-IRDye800CW demonstrated markedly different binding patterns, with all HNSCC cell lines showing robust uptake of the EGFR-targeted agent. Mean fluorescence intensity values for panitumumab-IRDye800CW are shown in Fig. 2B

### *In Vivo *Fluorescence Imaging and Biodistribution Analysis

*In vivo* imaging studies using HNSCC xenograft models confirmed the poor tumor targeting observed in *in vitro* studies. Whole-body fluorescence imaging at 2 (Supplementary Fig. 1) and 24 h post-injection revealed minimal tumor accumulation of pafolacianine in both FaDu and UMSCC47 xenograft models.

**Fig. 3:**
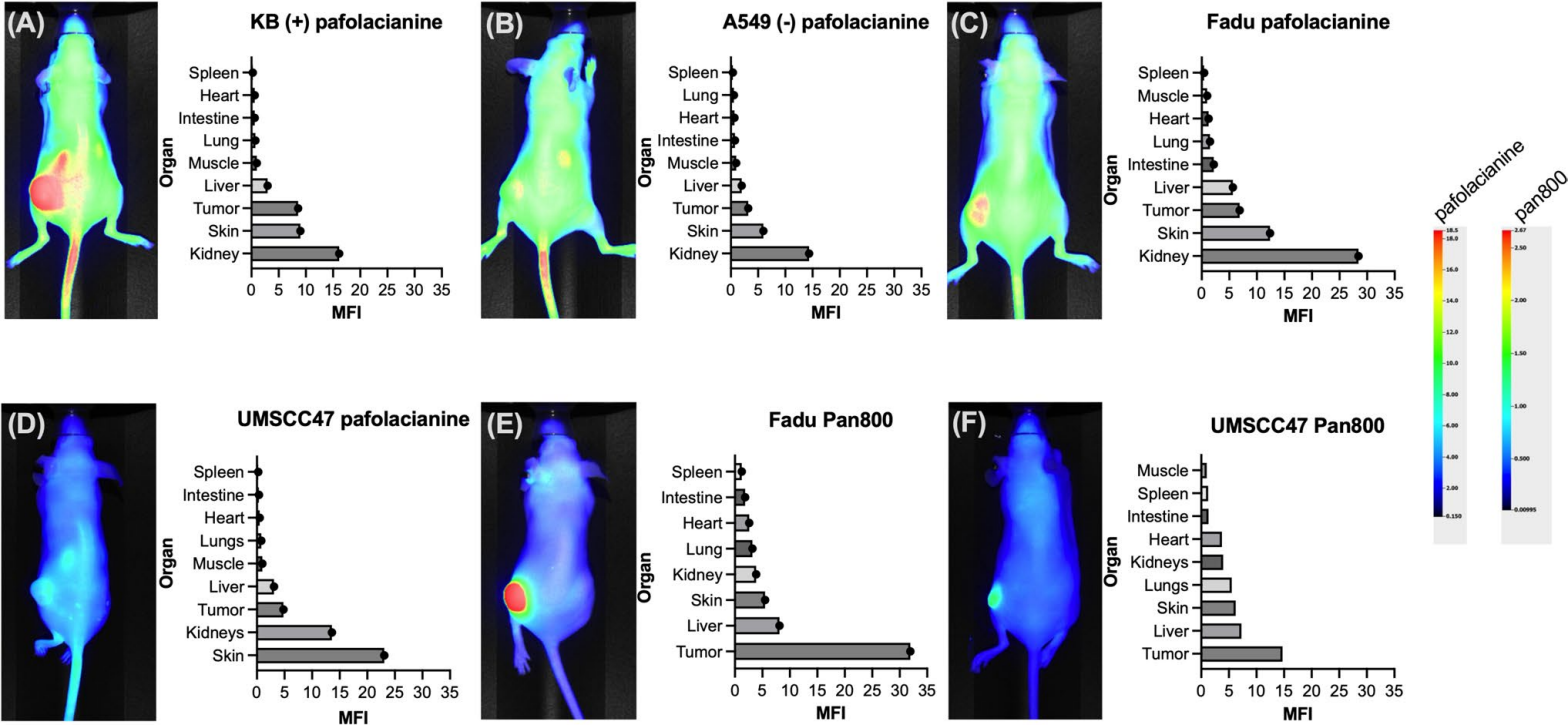
*In vivo* and ex vivo drug distribution for pafolacianine and Pan800. Corresponding contrast scales are given to the right. **(A-D)** *In vivo* images and ex vivo MFI of mice treated with pafolacianine after 24 h. **(E–F)** *In vivo* images and ex vivo MFI of mice treated with Pan800 after 72 h

Ex vivo tissue analysis confirmed the *in vivo* imaging findings and provided additional quantitative data on pafolacianine distribution. Fluorescence quantification revealed highest accumulation in kidneys (consistent with renal clearance of folate-based compounds), followed by skin and liver tissues. Tumor tissue fluorescence levels ranked among the lowest of all analyzed tissues. Qualitative assessment of ex vivo fluorescence distribution showed predominant accumulation in non-target tissues, particularly skin and kidneys (mode of excretion), with limited tumor-specific localization. Skin MFI values were significantly higher than tumor values in both xenograft models: FaDu skin (12.4 ± 1.8, *p* < 0.01 vs. tumor) and UMSCC47 skin (12.1 ± 1.6, *p* < 0.01 vs. tumor) (Fig. 3A-D).

Time-course analysis revealed no improvement in tumor targeting over extended observation periods. MFI values remained consistently low across all timepoints (2–24 h), with no evidence of the delayed tumor accumulation sometimes observed with imaging agents.

### Comparative Analysis with Panitumumab-IRDye800CW

Direct comparison with panitumumab-IRDye800CW provided important context for interpreting the pafolacianine results and validated our experimental approach. *In vivo* imaging of HNSCC xenografts treated with panitumumab-IRDye800CW demonstrated robust tumor targeting with excellent contrast (Fig. 3E-F). The biodistribution pattern of panitumumab-IRDye800CW showed preferential tumor accumulation with minimal background fluorescence in non-target tissues. The success of EGFR targeting reflects the well-documented overexpression of EGFR in 80–90% of HNSCC cases, while the failure of FR-α targeting aligns with our immunohistochemical findings of limited and heterogeneous FR-α expression in HNSCC specimens.

### Western Blot Analysis of Receptor Expression

Western blot analysis of whole cell lysates provided additional molecular evidence supporting our functional studies. FR-α protein expression was barely detectable in most HNSCC cell lines, with only faint bands observed even after extended exposure times. Densitometric analysis revealed FR-α expression levels that were 15–20 fold lower than the KB positive control cell line and comparable to the A549 negative control (Fig. 1B). Although supportive, it should be noted that expression by IHC or Western blot does not accurately measure the activity or turnover of the receptor which may result in higher levels of endocytosis.

## Discussion

This preclinical evaluation provides definitive evidence that pafolacianine exhibits limited utility for fluorescence-guided surgery in head and neck squamous cell carcinoma. Our multi-modal investigation, encompassing *in vitro* binding studies, *in vivo* imaging assessment, and clinical tissue analysis, demonstrated poor FR-α-mediated targeting. The observed MFI fall substantially below established thresholds for clinical utility, indicating that pafolacianine would not provide adequate tumor contrast for reliable intraoperative guidance in HNSCC procedures. Furthermore, the preferential accumulation in non-target tissues, particularly skin, would create significant background fluorescence that could interfere with surgical decision-making and potentially lead to false-positive identification of non-neoplastic tissue.

The biological basis for these negative findings lies in the fundamental difference in FR-α expression patterns between HNSCC and the cancer types where pafolacianine has demonstrated clinical success. Our immunohistochemical analysis revealed that only 12.5% of HNSCC specimens exhibited high FR-α expression (scores 6–9), with the majority showing low expression levels comparable to normal tissue. This finding contrasts markedly with ovarian cancer, where FR-α overexpression is observed in 80–90% of cases and serves as the biological foundation for pafolacianine's clinical efficacy [11]. The contrast between our pafolacianine results and the established success of panitumumab-IRDye800CW in HNSCC provides important insights into the biological requirements for effective fluorescence-guided surgery. The robust tumor targeting achieved with panitumumab-IRDye800CW demonstrates that clinically meaningful fluorescence contrast is achievable in HNSCC when appropriate molecular targets are selected [12, 13].

The publication of these negative findings serve a critical value by educating researchers in the field, "negative results are a crucial part of the educational learning process and selection of the most effective treatment" [14]. Our comprehensive evaluation provides convincing evidence that will prevent other research groups from pursuing similar investigations, thereby avoiding duplication of effort and misallocation of resources.

While our study provides comprehensive evidence regarding pafolacianine's limited utility in HNSCC, we recognize the use of established cell lines and xenograft models, while standard in preclinical research, may not fully recapitulate the biological complexity of human HNSCC. Patient-derived xenograft models or organoid systems might provide additional insights into the heterogeneity of FR-α expression observed in clinical specimens. The sample size of clinical specimens (*n* = 8 tumor, *n* = 8 normal) represents a limitation that could be addressed through larger, multi-institutional studies. Additionally, our *in vitro* experiments were designed to display a trend at multiple doses and time-points, and adding replicates can ensure statistical relevance. However, the consistency of low FR-α expression across our cohort, combined with the lack of significant differences between tumor and normal tissue, suggests that larger sample sizes would be unlikely to alter our fundamental conclusions.

## Conclusions

This comprehensive preclinical evaluation provides definitive evidence that pafolacianine exhibits limited utility for fluorescence-guided surgery in HNSCC due to insufficient folate receptor-α expression and poor tumor-to-background contrast.

## Supplementary Information

Fig. 4:  In vivo and ex vivo MFI of pafolacianine after 2 hours in (A) A549 (B) FaDu (C) UMSCC47 xenografts. (PNG 877 KB)

High resolution image (TIF 1.18 MB)

## Data Availability

Raw data supporting the conclusions of this article are available from the corresponding author upon reasonable request, in accordance with institutional data sharing policies and patient privacy protections.

## References

[1] Kashiwagi S, Choi HS (2023) Ovarian cancer-targeted near-infrared fluorophores for fluorescence-guided surgery. Ann Transl Med 11(6):274. 10.21037/atm-22-6455 37082670 10.21037/atm-22-6455 PMC10113083

[2] Fayaz M, Abbasher Hussien Mohamed Ahmed K (2023) Advancing intraoperative tumour detection and molecular-guided precision surgery, FDA’s approval of pafolacianine injection: an editorial. Ann Med Surg 86(1):18–19. 10.1097/MS9.0000000000001534 10.1097/MS9.0000000000001534 PMC10783271 38222777

[3] Dindere ME, Tanca A, Rusu M, Liehn EA, Bucur O (2022) Intraoperative tumor detection using pafolacianine. Int J Mol Sci 23(21):12842. 10.3390/ijms232112842 36361630 10.3390/ijms232112842 PMC9658182

[4] Gangadharan S et al (2021) Multiinstitutional phase 2 clinical trial of intraoperative molecular imaging of lung cancer. Ann Thorac Surg 112(4):1150–1159. 10.1016/j.athoracsur.2020.09.037 33221195 10.1016/j.athoracsur.2020.09.037 PMC10985531

[5] Li MM, Puram SV, Silverman DA, Old MO, Rocco JW, Kang SY (2019) Margin analysis in head and neck cancer: state of the art and future directions. Ann Surg Oncol 26(12):4070–4080. 10.1245/s10434-019-07645-9 31385128 10.1245/s10434-019-07645-9 PMC7382965

[6] Orosco RK et al (2018) Positive surgical margins in the 10 most common solid cancers. Sci Rep 8(1):5686. 10.1038/s41598-018-23403-5 10.1038/s41598-018-23403-5 PMC5890246 29632347

[7] Young K, Ma E, Kejriwal S, Nielsen T, Aulakh SS, Birkeland AC (2022) Intraoperative *in vivo* imaging modalities in head and neck cancer surgical margin delineation: a systematic review. Cancers (Basel) 14(1):3416. 10.3390/cancers14143416 35884477 10.3390/cancers14143416 PMC9323577

[8] Scaranti M, Cojocaru E, Banerjee S, Banerji U (2020) Exploiting the folate receptor α in oncology. Nat Rev Clin Oncol 17(6):349–359. 10.1038/s41571-020-0339-5 32152484 10.1038/s41571-020-0339-5

[9] Mahalingam SM et al (2018) Evaluation of novel tumor-targeted near-infrared probe for fluorescence-guided surgery of cancer. J Med Chem 61(21):9637–9646. 10.1021/acs.jmedchem.8b01115 30296376 10.1021/acs.jmedchem.8b01115

[10] Sansom O, Bogani D, Reichenbach L, Wells S (2024) Negative equity – the value of reporting negative results. Dis Model Mech 17(8):dmm050937. 10.1242/dmm.050937 39212951 10.1242/dmm.050937 PMC11381924

[11] Randall LM, Wenham RM, Low PS, Dowdy SC, Tanyi JL (2019) A phase II, multicenter, open-label trial of OTL38 injection for the intra-operative imaging of folate receptor-alpha positive ovarian cancer. Gynecol Oncol 155(1):63–68. 10.1016/j.ygyno.2019.07.010 31362825 10.1016/j.ygyno.2019.07.010

[12] Stone LD et al (2024) Interim phase II results using panitumumab-IRDye800CW during transoral robotic surgery in patients with oropharyngeal cancer. Clin Cancer Res 30(18):4016–4028. 10.1158/1078-0432.CCR-24-0940 39012279 10.1158/1078-0432.CCR-24-0940 PMC11398989

[13] Youn GM et al (2022) The use of panitumumab-IRDye800CW in a novel murine model for conjunctival squamous cell carcinoma. Translational Vision Science, Technology 11(7):23. 10.1167/tvst.11.7.23 35895055 10.1167/tvst.11.7.23 PMC9344218

[14] Nardo M, Guven DC, Yikilmaz AS, Singh S, Ahmed J (2023) Learning from failure: negative trials in oncology. J Immunother Precis Oncol 6(2):59–60. https://pmc.ncbi.nlm.nih.gov/articles/PMC10195015/. Accessed 29 July 2025 10.36401/JIPO-23-X1 PMC10195015 37214208

