# Supplementary figures and images for: Evaluation of Pafolacianine (Cytalux®) for Fluorescence-Guided Surgery in Head and Neck Squamous Cell Carcinoma: A Negative Study with Important Clinical Implications
Source: Mol Imaging Biol. 2025 Dec 11;28(1):106–15. doi: 10.1007/s11307-025-02068-3 (PMC12966201; doi:10.1007/s11307-025-02068-3)

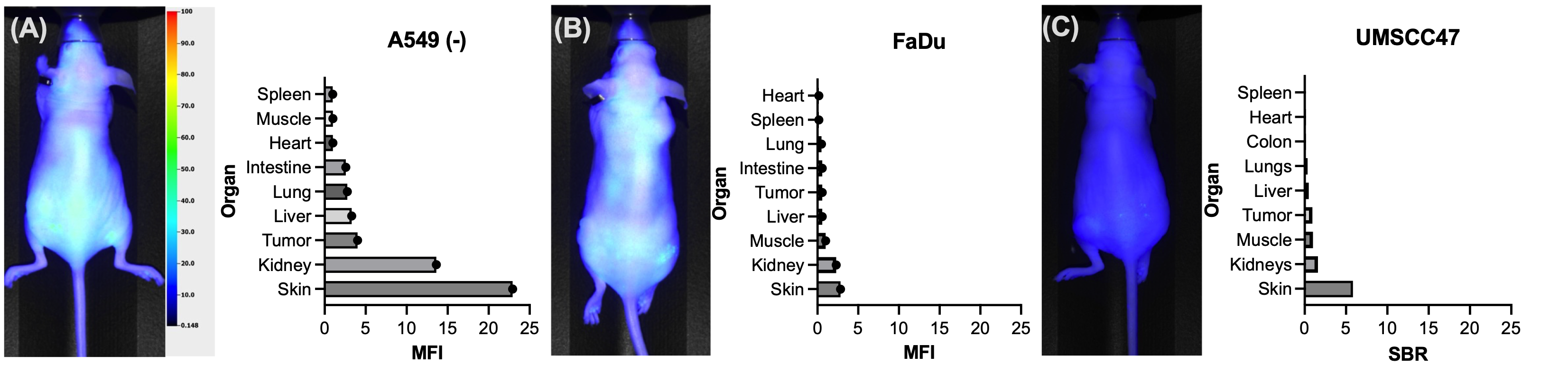

Supplement: Supplementary file 1 — In vivo and ex vivo MFI of pafolacianine after 2 hours in (A) A549 (B) FaDu (C) UMSCC47 xenografts. (PNG 877 KB) [file 11307_2025_2068_Fig4_ESM.png]

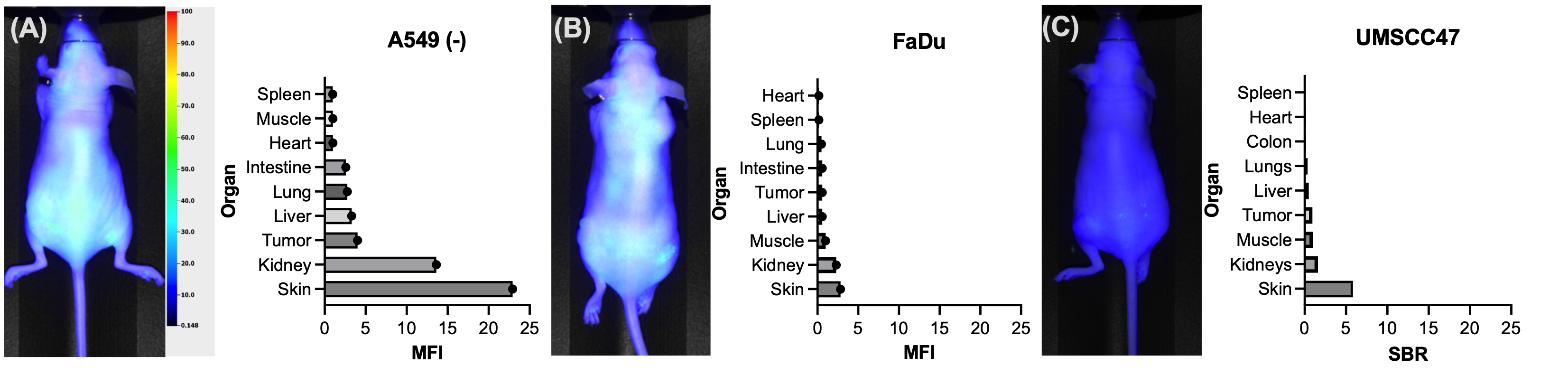

Supplement: Supplementary file 2 — High resolution image (TIF 1.18 MB) [file 11307_2025_2068_MOESM1_ESM.tiff]
